# First detection of *Theileria parva *in cattle from Cameroon in the absence of the main tick vector *Rhipicephalus appendiculatus*

**DOI:** 10.1111/tbed.13425

**Published:** 2020-03-16

**Authors:** Barberine A. Silatsa, Gustave Simo, Naftaly Githaka, Rolin Kamga, Farikou Oumarou, Christian Keambou Tiambo, Eunice Machuka, Jean‐Baka Domelevo, David Odongo, Richard Bishop, Jules‐Roger Kuiate, Flobert Njiokou, Appolinaire Djikeng, Roger Pelle

**Affiliations:** ^1^ Biosciences Eastern and Central Africa ‐ International Livestock Research Institute (BecA‐ILRI) Hub Nairobi Kenya; ^2^ Molecular Parasitology and Entomology Unit Department of Biochemistry Faculty of Sciences University of Dschang Dschang Cameroon; ^3^ Department of Biosciences International Livestock Research Institute (ILRI) Nairobi Kenya; ^4^ Special Mission for Eradication of Tsetse Flies Regional tsetse Division of Adamawa MINEPIA Adamawa Cameroon; ^5^ School of Biological Sciences University of Nairobi Nairobi Kenya; ^6^ Veterinary Microbiology and Pathology (VMP) Washington State University Pullman WA USA; ^7^ Department of Biochemistry Faculty of Sciences University of Dschang Dschang Cameroon; ^8^ Laboratory of General Biology Faculty of Sciences University of Yaounde I Yaounde Cameroon; ^9^ Present address: Department of Biochemistry Faculty of Sciences University of Dschang Dschang Cameroon; ^10^ Present address: Centre for Tropical Livestock Genetics and Health The University of Edinburgh Edinburgh UK

**Keywords:** Cameroon, cattle, East Coast fever, identification, *Theileria parva*

## Abstract

A major risk factor for the spread of livestock diseases and their vectors is the uncontrolled transboundary movement of live animals for trade and grazing. Such movements constrain effective control of tick‐transmitted pathogens, including *Theileria parva*. Only limited studies have been undertaken to identify ticks and tick‐borne diseases (TTBDs) affecting cattle in central African countries, including Cameroon. We hereby report the collection of baseline data on the prevalence of *T. parva* in Cameroon through a countrywide cross‐sectional survey, conducted in 2016, involving collection of blood samples from cattle from 63 sites across the five agro‐ecological zones (AEZs) of the country. ELISA‐based surveillance of infected cattle was performed on 479 randomly selected samples and revealed specific antibodies to *T. parva *in 22.7% and *T. mutans* in 41.1% of cattle*. *Screening of 1,340 representative DNA samples for the presence of *T. parva* identified 25 (1.86%) positives using a p104 antigen gene‐based nested PCR assay. The positives were distributed across agro‐ecological zones I, II, III and V. None of the p104 positive cattle exhibited clinical symptoms of East Coast fever (ECF). Using reverse line blot (RLB), 58 (4.3%) and 1,139 (85%) of the samples reacted with the *T. parva* and *T. mutans* oligonucleotide probes, respectively. This represents the first report of *T. parva* from Cameroon. Surprisingly, no *Rhipicephalus appendiculatus* ticks, the main vector of *T. parva*, were identified in a parallel study involving comprehensive morphological and molecular survey of tick species present in the country. Only two of the 25 p104 positive cattle were PCR‐positive for the CD8+ T‐cell target schizont‐expressed antigen gene Tp1. Cloning and sequencing of Tp1 amplicons revealed sequence identity with the reference *T. parva *Muguga. This new finding raises serious concerns of a potential spread of ECF into the central African region.

## 1 INTRODUCTION

Theilerioses are tick‐borne protozoan diseases that cause major livestock production losses in tropical regions of the world (Jongejan & Uilenberg, 1994). Losses are attributable to a combination of mortality, decreased meat and milk production and the cost of treatment (Norval, Perry, & Young, 1992). *Theileria parva*, *T. annulata*, *T. taurotragi*, *T. mutans*, *T. Velifera *and *T. orientalis/T. buffeli* are species known to infect cattle, but with different levels of pathogenicity (Morzaria, 1989). *Theileria mutans* is typically benign compared to the highly virulent *T. parva* (Flanagan & Le Roux, 1957). However, recent observations involving multiple *Theileria* infections in cattle have demonstrated that when an animal is first infected by less pathogenic *Theileria* species, specifically, *T. mutans and T. velifera*, the subsequent *T. parva* infection is frequently asymptomatic. One hypothesis is that heterologous protection helps these animals to survive subsequent *T. parva* infections (Woolhouse et al., 2015).

The most economically important *Theileria* infection in sub‐Saharan Africa is East Coast fever (ECF). ECF is a lymphoproliferative disease caused by *T. parva* infections. This fatal disease kills over one million cattle each year in sub‐Saharan Africa, resulting in severe economic losses for smallholder farmers and pastoralists (Nene et al., 2016; Norval et al., 1992). The principal vector *Rhipicephalus appendiculatus* is a three‐host tick whose distribution coincides with that of clinical ECF (Young & Leitch, 1981). Other confirmed vectors are *R. zambeziensis* in parts of Southern Africa and *R. duttoni* in Angola. Additionally, other *Rhipicephalus* and *Hyalomma* species have been shown experimentally to be potential vectors of *T. parva* group parasites (Norval et al., 1992; Theiler, 1909; Uilenberg, 1981). Among these species, *R. capensis, R. evertsi, R. simus, H. dormedarii and H. impressum* have been reported to infest bovines in Cameroon, although the main vector, *R. appendiculatus,* has not yet been reported in the country (Morel & Magimel, 1959; Morel & Mouchet, 1958; Rageau, 1953).

The reported range of *T. parva* infection extends northward from eastern South Africa to southern Sudan, and westward from eastern Kenya and Tanzania to eastern Democratic Republic of Congo, covering 15 countries (Nene et al., 2016; Norval et al., 1992; Olwoch, Reyers, Engelbrecht, & Erasmus, 2008). The principal ECF control method used currently is the application of acaricide to kill the tick vector, but this is not sustainable in the medium term, due to emerging resistance and concerns regarding environmental contamination and presence of drug residues in the food chain (De Meneghi, Stachurski, & Adakal, 2016; Gachohi, Skilton, Hansen, Ngumi, & Kitala, 2012; Graf et al., 2004). Immunization of cattle with live *T*. *Parva* sporozoites and treatment with a long‐acting formulation of oxytetracycline is being increasingly adopted (Brown et al., 1977; Di Giulio, Lynen, Morzaria, Oura, & Bishop, 2009; Nene et al., 2016). This ‘infection and treatment method’ (ITM) of vaccination results in an asymptomatic or mild episode of ECF, which induces long‐term immunity to the disease in cattle. ITM induces solid protection especially against homologous parasite challenge, believed to be at least partially mediated by CD8^+^ T cells (McKeever et al., 1994). ITM sometimes fail to protect especially against challenge with buffalo derived parasites (Sitt et al., 2015), and if the vaccine is not correctly administered animals can succumb to severe disease (Irvin & Mwamachi, 1983). Moreover, since it typically induces a tick‐transmissible carrier state, ITM can facilitate the spread of the parasite through importation of vaccinated cattle (De Deken et al., 2007; Di Giulio et al., 2009). There are ongoing efforts to develop next generation subunit vaccines but these are still some way from proof of concept in field trials (Nene et al., 2016).

*Theileria parva* infections may be present in Cameroon since reports of cattle presenting clinical symptoms similar to those observed in theileriosis have occasionally been noted (R. Pelle and B. A. Silatsa, personal communication). If this is the case, the probable route of introduction of *T. parva* is cross‐border livestock movement for trade, or transhumance. Indeed, Cameroon is located on a major cattle trade route between eastern and western Africa countries. Intra‐regional trade and importation of animals from outside the region, prolonged periods of drought and conflicts trigger very extensive migration of people and their livestock in the subregion (Bouslikhane, 2015; Di Nardo, Knowles, & Paton, 2011; Motta et al., 2017; Seignobos, 2008). Cross‐border livestock and wildlife movements are one of the most important drivers of the spread of ticks and tick‐borne‐pathogens globally. The problem is more serious in sub‐Saharan Africa (SSA) relative to other regions of the world, due to the lack of effective implementation of quarantine policies at the borders.

The recent deployment of the ITM live vaccine has been very effective in controlling disease in certain ECF endemic areas, particularly among pastoralists in Tanzania (Di Giulio et al., 2009), with measurable benefits to the local population (Marsh, Yoder, Deboch, McElwain, & Palmer, 2016). However, due to the long‐term carrier state induced by vaccination, non‐vaccinated cattle can be infected with vaccine component genotypes (Oura et al., 2007). This creates a risk of importing the parasite into areas previously free of infection, especially at the border of the existing distribution (McKeever, 2007). One illustration of this scenario is an outbreak of ECF in the Comoros following cattle importation from Tanzania where the disease is endemic (De Deken et al., 2007). Cameroon is potentially connected to the eastern African regions where ECF is endemic through cross‐border livestock movements (only one country, the Central African Republic, separates Cameroon from South Sudan, an ECF endemic country), and the national borders do not present a barrier to pathogen and disease dissemination (Motta et al., 2017).

Knowledge of the prevalence of ticks and tick‐borne diseases is a prerequisite to evaluation of the importance and risk of diseases and to design of effective control strategies. To the best of our knowledge*, T. parva* infections have not yet been documented in Cameroon. The incidence and prevalence of *T. parva* and ECF have not been documented, and the potential economic losses due to this parasite are unknown. The cross‐sectional survey reported in this paper aimed to assess the occurrence of *T. parva* infection among cattle in the five agro‐ecological zones (AEZs) of Cameroon in order to evaluate the potential risk of future ECF outbreaks in the central Africa region.

## 2 MATERIALS AND METHODS

### 2.1 Study areas

**Figure 1:**
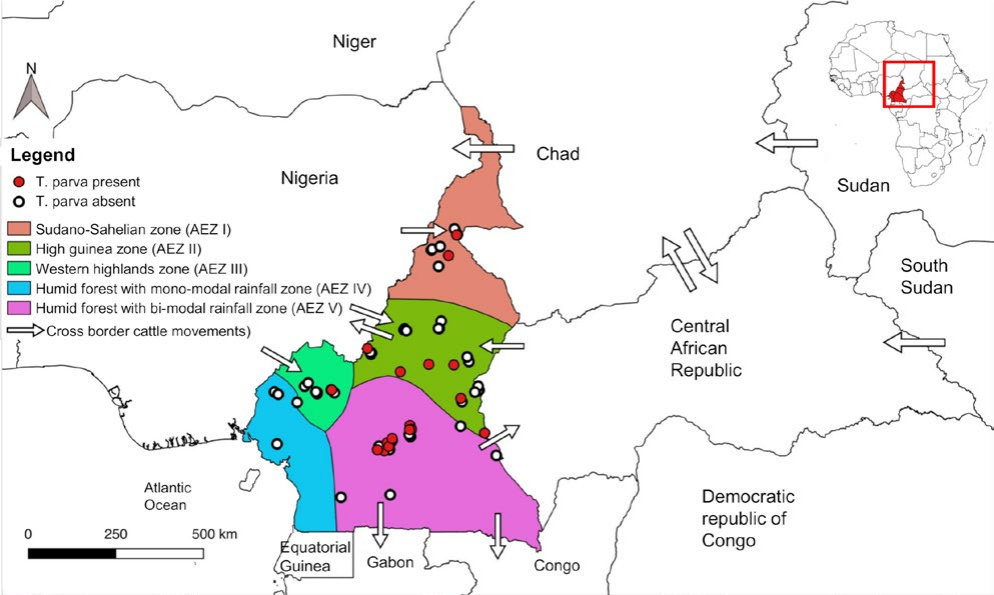
Map showing sampling sites as well as locations where samples were positive to *Theileria parva* p104 screening test in Cameroon

A cross‐sectional survey was conducted from April to August 2016 (during the wet season). Sampling was conducted at 63 sites across the five AEZs of Cameroon (Figure 1). The number of sites sampled in each zone was determined by livestock density and willingness of farmers to participate to the survey. Key geographic and climatic features of each AEZ have been previously described (Silatsa, Kuiate, et al., 2019a). AEZ II represents the main cattle production area in Cameroon with a population estimated at approximately 1.25 million head of cattle (Motta et al., 2017). This region is the main destination of transhumant herders originating from neighbouring countries (Motta et al., 2018). AEZ III mainly covers the west and the north‐west region and hosts approximately 610 000 head of cattle (Motta et al., 2017). Many farmers in AEZ V are refugees originating from conflict zones in Central African Republic (Seignobos, 2008).

Most cattle sampled in the different AEZs were of local breeds (B*os indicus*) with a few exotic (*Bos taurus*) or crossbreed (*taurus × indicus*) animals. Except for a minority of farms where a combination of stall feeding and free grazing is practiced, most cattle are reared under an open grazing system.

### 2.2 Cattle blood collection, sera preparation and DNA isolation

Blood samples were collected from jugular vein of 1,340 cattle in EDTA and plain vacutainer tubes of 5 ml. The samples were transferred to the Molecular Parasitology and Entomological Sub‐unit at the University of Dschang in a cool box and refrigerated. The next day, the sera were separated by centrifugation at 1,000 *g* for 20 min, aliquoted in 2‐ml sterile Eppendorf tubes and store at −20°C. Genomic DNA (gDNA) was extracted from whole blood using the DNeasy Blood & Tissue Kit (Qiagen) as recommended by the manufacturer. Purified gDNA and serum samples were transported to Biosciences eastern and central Africa (BecA) Hub at the International Livestock Research Institute (ILRI) in Nairobi for serological and molecular analyses.

### 2.3 Detection of antibodies to *T. parva* and *T. mutans* with ELISA test

ELISA test was performed only on a subset of samples. The resampling was performed in the programming software R, using the function ‘*sample’* with a bootstrapping selection, to ensure that the selection process was randomized*.* The random process used the primary sample unit (which was the herd in this case) as the stratification variable. All animals in each herd were sampled at 1/3 (about 33%) without replacement and concatenated to make an overall sample size for the study area. Using this method, 479 serum samples were randomly selected from a total of 1,340 samples.

Indirect ELISA tests were used to detect antibodies to *T. parva* and *T. mutans* using recombinant polymorphic immunodominant molecule (PIM) and p32 antigens, respectively (Katende, Goddeeris, Morzaria, Nkonge, & Musoke, 1990; Katende et al., 1998). Antibodies against these parasites were assessed with kits provided by ILRI according to the protocols described by Katende et al. (1998). Briefly, plates were coated with the appropriate diluted antigens; then, uncoated free sites were blocked to prevent non‐specific binding using a standard buffer. Test and control sera were diluted and added to the pre‐washed antigen coated plate in duplicate. After incubation with the serum, the plates were washed several times and the anti‐IgG/enzyme conjugate was added. Plates were then washed five times. Finally, the substrate was diluted and added in each well. After incubation, plates were read at 405 nm with the Immunoskan ELISA reader using program EDI. Data analysis was performed using the ELISA programme integrated into the ELISA reader, and the results were presented as percent positivity (PP). Predetermined positive and negative control sera were included in each ELISA test plate. Optical density (OD) readings from the reference positive control sera were used to compute the PP for the test sera (Wright, Nilsson, Rooij, Lelenta, & Jeggo, 1993). PP values of 20 and above were considered positive for *T. mutans* and *T. parva*.

### 2.4 *Theileria parva* screening in blood samples from cattle using p104 PCR test

*Theileria parva* prevalence was assessed from whole blood gDNA using the p104 nested PCR (nPCR) assay. This highly sensitive and specific assay targets the single copy gene encoding the *T. parva* 104 kDa rhoptry antigen. Oligonucleotide outer primers p104Fwd1 (5′‐ATTTAAGGAACCTGACGTGACTGC‐3′) and p104Rev1 (5′‐TAAGATGCCGACTATTAATGACACC‐3′) and inner primers p104Fwd2 (5′‐GGCCAAGGTCTCCTTCAGAATACG‐3′) and p104Rev2 (5′‐TGGGTGTGTTTCCTCGTCATCTGC‐3′) were used as previously described (Odongo, Sunter, Kiara, Skilton, & Bishop, 2010). The inner primer pair amplifies a conserved fragment of 278 bp of the p104 antigen gene spanning nucleotide 2,333 to nucleotide 2,610 of the 2,775 bp long open‐reading frame (ORF). Both PCRs were performed using AccuPower^®^ Taq 2× PCR Master Mix (Bioneer). The primary PCR contained 0.25 µM of forward and reverse primers, 20 ng of gDNA, and nuclease‐free water added to bring the reaction to a final volume of 20 μl. The PCR was performed using the GeneAmp^®^ PCR System 9,700 thermocycler (Applied Biosystems). For the first PCR, the thermal cycling programme consisted of an initial denaturation at 95°C for 3 min followed by 35 cycles of denaturation at 94°C for 1 min, an annealing at 60°C for 1 min and an extension at 72°C for 1 min. A final extension was performed at 72°C for 10 min.

One microlitre of the primary PCR product was used as template in the nested PCR. For this nested PCR, everything was the same as in the primary PCR except for the primers. A positive control and a negative control were included in the screening. The amplification conditions for the nested PCR were as described for the primary PCR, except that the annealing temperature was reduced to 55°C for 1 min.

Seven microlitre of PCR products were run on a 1.8% agarose electrophoresis gel containing GelRed (Biotium) and visualized under UV light.

### 2.5 PCR amplification of Tp1 gene locus

Tp1 is a *T*. *parva* antigen recognized by immune bovine cytotoxic T‐lymphocytes (Graham et al., 2006), encoded by a single copy gene containing a 1,632‐bp‐long ORF. The 405‐bp PCR‐amplified region encodes 134 amino acids containing the defined 11 amino acid‐long CD8^+^ T‐cell epitope (Pelle et al., 2011). Samples tested positive for p104 were screened for the presence of Tp1 gene in a nested PCR using the outer primers (Tp1‐Fw1 5′‐ATGGCCACTTCAATTGCATTTGCC‐3′ and Tp1‐ Rev1 5′‐TTAAATGAAATATTTATGAGCTTC‐3′) and the inner primers (5′‐TGCATTTGCCGCTGATCCTGGATTCTG‐3′ and 5′‐TGAGCTTCGTATACACCCTCGTATTCG‐3′) as previously described (Salih et al., 2017). PCR conditions for the first and the second round of Tp1 gene amplification were as follows: 95°C for 5 min followed by 40 cycles of 95°C for 30 s, 55°C for 30 s, 72°C for 1 min and a final extension of 9 min at 72°C. Seven microlitres of Tp1 nested PCR products were analysed by electrophoresis in a 1.8% agarose gel. The second PCR was repeated for positive samples in order to increase the quantity of amplified products. The amplified products from each sample were pooled and purified using the QIAquick PCR Purification Kit (Qiagen) following the manufacturers’ protocol.

### 2.6 Sequencing of p104 and Tp1 gene loci

To increase the quantity of amplified products for sequencing purposes, nested PCR was repeated for positive samples to p104 test. Amplified products from the same samples were pooled and purified using the QIAquick PCR Purification Kit (Qiagen) following the manufacturers’ protocol. Four microlitre of PCR products were run on a 1.8% agarose gel to check the quality of the amplicons. The final concentration of purified PCR product was determined with a spectrophotometer (WPA Lightwave II). The purified amplicons were sequenced at Bioneer, using forward and reverse inner primers used during the nested PCR.

For Tp1 gene, purified PCR product was cloned using InsTAclone PCR product cloning kit (MBI Fermentas), according to manufacturer's protocol and sequenced by Sanger method using M13/pUC primers.

### 2.7 Sequences analysis and phylogenetic reconstruction

Sequences obtained from PCR‐positive samples were manually edited and assembled, and consensus sequences were generated and aligned using CLC Main Workbench software v7.8.1 (CLC bio). BLAST search was performed to confirm species identity of these sequences compared with available sequences in the GenBank database. Variants generated in the present study were compared to reference sequences available in the GenBank. Phylogenetic reconstruction was performed on p104 amino acid sequences without the flanking primer regions removed. DNA sequences were translated into proteins using CLC work bench 8.0 and aligned using MEGA v7.0. The phylogenetic trees were built using a hierarchical likelihood ratio test based on the lowest Bayesian information criterion using MEGA v7.0. To find the evolutionary substitution model that best describes the evolution of p104, the neighbor joining trees were plotted using 1,000 bootstrap replicates.

### 2.8 PCR amplification of the 18S RRNA gene of Theileria/Babesia

The primers RLB F2 (5′‐GAC ACA GGG AGG TAG TGA CAA G‐3′) and RLB R2 (5′‐Biotin‐CTA AGA ATT TCA CCT CTA ACA GT‐3′) that are specific for the *Theiler*ia and *Babesia* were used to amplify the hypervariable (V4) region of parasite 18S rRNA gene using a touch‐down PCR recipe as previously described, with slight modifications (Nijhof et al., 2003). The PCR mixture consisted of 12.5 μl of AccuPower^®^ Taq 2× PCR Master Mix (Bioneer), 1 μl of each primer (10 pmol) and 50 ng/μl of gDNA. The final volume was made up to 25 μl with molecular biology grade water. Positive and negative controls were included in the assay. The amplification steps for 18S rRNA coding gene of *Theileria/Babesia* consisted of preheating at 94°C for 5 min, 10 cycles of 94°C for 20 s, 67°C for 30 s and 68°C for 30 s with annealing temperature decreasing at every cycle by 1°C until 57°C. This was followed by 30 cycles of 94°C for 20 s, 57°C for 30 s and 68°C for 30 s. The reactions were cycled using the GeneAmp^®^ PCR System 9700 (Applied Biosystems) with heated lid option.

### 2.9 Reverse line blot hybridization

Reverse line blot (RLB) hybridization was performed against *T. parva* and *T. mutans* probes as previously described with modifications (Gubbels et al., 1999). A Biodyne‐C membrane (Pall Life Sciences) was activated by incubating in 16% 1‐ethy‐3‐(3‐dimethyl‐amino‐propyl) carbodiimide (EDAC) (Sigma‐Aldrich) at room temperature for 10 min. The membrane was then washed with double distilled water for about 2 min and placed on a screen‐blotter (Sanplatec). Oligonucleotide probes covalently linked with an N‐(monomethoxytrityl(MMT))‐C6 amino linker were obtained from Hokkaido System Science, Japan, and reconstituted as 100 pmol stocks. Ten microlitres of stock probes were diluted with 0.5 M NaHCO3 to final volume of 120 μl and bound on the activated membrane by incubating for 5 min at room temperature. The membrane was inactivated with 100 mM NaOH for exactly 8 min at room temperature. The membrane was then washed with 100 ml of 2x SSPE/0.5% sodium dodecyl sulphate (SDS) for 5 min at 60°C with gentle shaking. The membrane was either used immediately or stored at 4°C in a sealed plastic containing 20 mM EDTA, pH 8. A volume of 20 μl of the PCR product was diluted to a final volume of 120 μl with 2x SSPE/0.1% SDS and denatured by heating at 99°C for 10 min on a thermocycler, and immediately snap‐cooled on ice. The denatured products were applied on the pre‐prepared membrane containing DNA probes and incubated for 60 min at 42°C. The membrane was washed twice using 2x SSPE/0.5% SDS buffer at 52°C for 10 min. The membrane was then incubated with IRdye 680LT (1/5,000 dilution) in 10 ml 2× SSPE/0.1% SDS with 0.2% Tween 20 and then visualized with ODYSSEY LICOR infrared imaging system.

### 2.10 Statistical analysis

P104 PCR, ELISA and RLB data were used to estimate the population prevalence of tick‐borne infections at 95% confidence intervals. The data were analysed using R (R Core Team, 2010) at 5% level of significance. The strength of association between agro‐ecological zones and the p104 prevalence was estimated by odds ratios (OR). A multivariate mixed logistic regression model was used to estimate the odds ratio for the risk of *T. parva* infection with adjustment for agro‐ecological zone. To assess the goodness‐of‐fit of the logistic models, the Hosmer–Lemeshow test was used to determine whether or not the observed outcome rates match the expected rates in different sub‐groups (agro‐ecological zones).

## 3 RESULTS

### 3.1 Serological analysis

**Figure 2:**
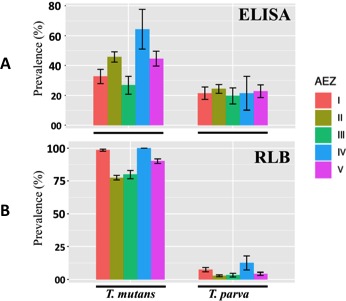
*Theileria parva* and *T. mutans* prevalence from ELISA test (a) and RLB test (b). AEZ: Agro‐ecological zones; I, II, III, IV and V represent different AEZ; the bars are confidence intervals at 95%; values are statistically significant at *p* < .001

Seroprevalence of *T. parva* and *T. mutans* was assessed using the ELISA tests. The overall mean antibody prevalence was 22.75% for *T. parva*. The average serum antibody prevalence of *T. parva* was relatively constant in all AEZs. It was 21.42%, 24.28%, 19.64%, 21.42% and 22.77% for AEZ I, II, III, IV and V, respectively, with PP values ranging from 20 to 84 relative to the positive control. Overall *T. mutans* serum antibody prevalence was higher (41.12%) than *T. parva.* In addition, antibody prevalence was higher in AEZ IV than in the other AEZs (Figure 2b).

### 3.2 Molecular detection of *Theileria parva* in blood samples from cattle

A nested PCR targeting p104 gene was used to determine the prevalence of *T. parva* infection across the 5 AEZs. Of the 1,340 samples analysed, 25 (1.86%) were positive for p104 PCR screening test. Positives animals were found in 17 of 63 sampling sites and distributed in four of five AEZs as shown in the Table 1. *T. parva* infection was significantly higher in AEZ V compare to the other AEZs (4.92% (95% CI: 2.94–8.15, *p* < .05)).

**Table 1:** Prevalence and distribution *Theileria parva* infections from p104 PCR test across agro‐ecological zones of Cameroon

Agro‐ecological zones	Divisions sampled	Number of sites	Number of sample	Number of positive	Prevalence % (95% CI)	Odd ratio	*p*‐value
AEZ I	Mayo‐Louti, Benoué	9	254	2	0.78 (0.19–3.09)	Reference	–
AEZ II	Mayo‐Banyo, Djérem, Mbere, Vina, Faro‐et‐Deo, Lom‐et‐Djerem	22	603	8	1.32 (0.66–2.63)	1.69	.507
AEZ III	Bamboutos, Noun, Lebialem, Mezam	10	159	1	0.62 (0.08–4.32)	0.79	.854
AEZ IV	Manyu, Meme	3	40	0	0	–	–
AEZ V	Haute sanaga, Mvila, Dja‐et‐Lobo, Kadey	19	284	14	4.92 (2.94–8.15)	6.53	.014

### 3.3 Sequencing and phylogenetic analysis

The two chromatograms from each individual (forward and reverse sequences) amplicon were edited manually, and a consensus sequence was generated. Good quality consensus sequences were obtained from 18 of the 25 isolates analysed. The final sequence length determined for p104 gene fragments was 276 base pairs. BLAST analysis of these sequences revealed a high identity value (ranging from 98% to 100%) relative to p104 sequences of *T. parva* available in the GenBank database. The p104 PCR and sequencing analysis confirmed the presence of *T. parva* infection in Cameroon.

The eighteen consensus p104 sequences obtained in the present study were collapsed into nine (H1 to H9) genotypes. H1 was the most predominant genotype, identified in 8 of 18 isolates. H7 and H9 were each represented in two isolates, while the remainder were represented by a single isolate. When these genotypes in the Cameroonian parasites were compared with the previously identified allele 1 initially identified in the Muguga stock, they were differentiated by a total of 14 single nucleotide polymorphic sites. The novel p104 gene sequence identified from this study were deposited in the NCBI GenBank database (GenBank accession numbers MK568798‐MK568804; Data S1).

**Figure 3:**
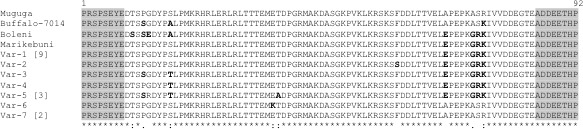
CLUSTAL multiple amino acid sequences alignments of p104 antigen variants. The single letter amino acid code is used throughout. Variants named Var_1‐Var_7. Residues conserved in all sequences are identified below the alignment (*). Brackets indicate the number of samples in which the variant was observed

Analysis of the predicted amino acid sequences of the 18 p104 sequences yielded seven different variants (Var1 to Var 7). All the sequences containing the flanking primer regions were aligned; then, the primers regions were trimmed prior to analysis. There were substitutions in 11 of the 76 amino acids comprising this p104 protein fragment. However, no indel was observed. When compared with the known variants (Iams, Hall, Webster, & Musoke, 1990; Skilton, Bishop, Katende, Mwaura, & Morzaria, 2002), genetic distances between p104 amino acid sequences ranged from 0 (Muguga and Var 7 [this study]), Marikebuni and Var 1 [this study]), to 10.47 (Boleni compared with Var 6, and also Var 5 and Var 6 [This study]) (Table 2, Figure 3).

**Table 2:** Genetic distance between p104 protein variants

Muguga^a^	Buffalo^a^	Boleni	Marike^a^	Var_1	Var_2	Var_3	Var_4	Var_5	Var_6	Var_7	
0	3.49	9.3	4.65	4.65	5.81	6.98	5.81	9.3	1.16	**0**	Muguga
	0	5.81	5.81	5.81	6.98	4.65	5.81	6.98	4.65	3.49	Buffalo_7014
		0	4.65	4.65	5.81	3.49	4.65	4.65	10.47	9.3	Boleni
			0	**0**	1.16	2.33	1.16	4.65	5.81	4.65	Marikebuni
				0	1.16	2.33	1.16	4.65	5.81	4.65	Var_1
					0	3.49	2.33	5.81	6.98	5.81	Var_2
						0	1.16	2.33	8.14	6.98	Var_3
							0	3.49	6.98	5.81	Var_4
								0	10.47	9.3	Var_5
									0	1.16	Var_6
										0	Var_7

Note

p104 variant 7 and variant 1 are identical to the Muguga and Marikebuni antigens, respectively. Bold values were used to highlight p104 protein variant that are hundred percent identical to vaccine strains (Muguga and Marikebuni). That is genetic distance = 0.

^a^ Muguga, *T. parva* stabilate 73; Buffalo, *T. parva* strain Buffalo‐7014; Marike, *T. parva* strain Marikebuni 3014.

Var 1 was the most predominant genotype (9 isolates), followed by Var 5 and Var 7 represented by 3 and 2 isolates, respectively. The rest were each represented each by a single sample. Comparative analysis with the amino acid sequences of the four known p104 variants previously reported in cattle and buffalo revealed that Var 1 and Var 7 were identical to Marikebuni and Muguga stocks, respectively.

**Figure 4:**
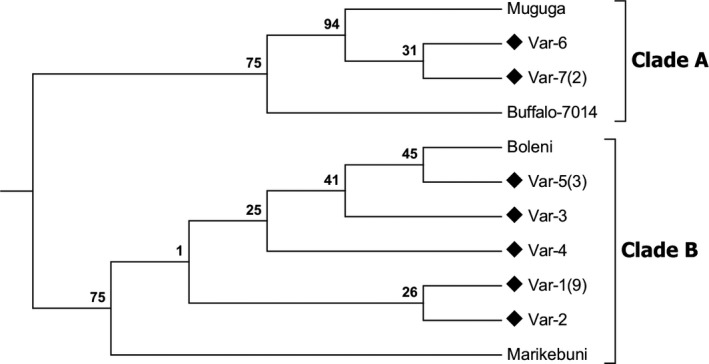
Phylogenetic relationship of *Theileria parva* strains as revealed by p104 amino acid sequence analysis. The evolutionary history was inferred using the neighbor joining method. The percentage of replicate trees in which the associated taxa clustered together in the bootstrap test (1,000 replicates) are shown above the branches. The evolutionary distances were computed using the Poisson correction method. The rate variation among sites was modelled with a gamma distribution (shape parameter = 5). There was a total of 86 positions in the final data set

For phylogenetic analysis, the predicted amino acid sequences from this study were compared with the four known p104 variants previously reported from East Africa. Neighbor joining (NJ) tree analysis resulted in a topology with relatively high bootstrap support values at the majority of nodes. Sequences obtained in this study could be grouped into 2 clades. Clade A comprises Var 7 and Var 6 together with the Muguga and Buffalo‐7014 stocks, whereas Var 1, 2, 3, 4 and 5 clustered with Boleni and Marikebuni stocks (Figure 4 **).**

We succeeded in amplification of a section of the Tp1gene for 2 of the 25 p104 positive isolates. These were cloned and sequence. Sequences analysis revealed only a single antigen variant. The CD8^+^ T‐cell epitope variant observed was identical to the one in the Muguga cocktail live vaccine (VGYPKVKEEML).

### 3.4 RLB analysis

RLB analysis was undertaken on 1,340 samples (Figure 2a). 58 (4.3%) and 1,139 (85%) of the samples reacted with the *T. parva* and *T. mutans* oligonucleotide probes, respectively. The prevalence of each of the haemoparasites within each AEZ is shown in Figure 2c. We investigated how frequently co‐infections occurred involving the two *Theileria* species. The results indicate that overall percentage of samples that were detected with both *T. parva* and *T. mutans* probes was 4.2%. *T. parva* was detected as a mixed infection with *T. mutans* in 98.3% of *T. parva* positive samples (57 of 58).

## 4 DISCUSSION

The present study was carried out to investigate the possible presence of *T. parva* infection in cattle in the five major agro‐ecological zones of Cameroon. The overall prevalence of *T. parva* based on p104 PCR, serology and RLB tests was low when compared to studies using the same tests in ECF endemic countries (Gachohi et al., 2012; Kerario et al., 2017; Njiiri et al., 2015).

*Theileria parva* prevalence based on the p104 PCR test was variable between agro‐ecological zones (0%–4.92%) with most of the infections detected in cattle from AEZs V and II. Agro‐ecological zones II and V are the main destination of herds resulting from transhumance, or originating from conflict zones in neighbouring countries (Motta et al., 2017; Seignobos, 2008). This could explain why most infections were detected in these areas.

The cattle sampled in the present study were apparently healthy and presented no clinical signs of ECF. Nonetheless, infection of cattle with *T. parva* was confirmed using three different tests. This significant finding suggests that cattle in Cameroon are subclinical carriers of *T. parva* parasite. This is of great potential epidemiological importance since infected cattle may act as a reservoir for transmission of the disease in future if a competent tick vector either colonizes Cameroon from an adjacent country, or a novel tick species evolves the capacity to transmit *T. parva*. Fortunately, the major *T. parva* tick vector *R. appendiculatus* was not found in Cameroon, during a recent comprehensive survey of ticks present in all agro‐ecological zones in the country (Silatsa, Simo, et al., 2019b). It is, however, possible that other *Rhipicephalus* and *Hyalomma* species that are present in Cameroon for which the ability to transmit *T. parva* infection has been demonstrated experimentally could potentially evolve to become vectors in future (Norval et al., 1992; Theiler, 1909; Uilenberg, 1981). *T. parva* transmission may occur within Cameroon since reports of cattle presenting clinical symptoms similar to those observed in theileriosis have occasionally been noted (R. Pelle and B. A. Silatsa personal communication). Approximately 23% of the sampled cattle were serologically positive for *T. parva*. If there is no *T. parva* transmission occurring on the field, this high level of seroprevalence suggests that approximately 23% of the cattle population in Cameroon is imported from eastern African countries. This scenario seems unlikely, although the nature of transboundary cattle movement means that there are no detailed official records available. The data suggest that at least some transmission may occur in the field. However, this needs to be demonstrated by additional studies. One important area for future investigation will to assess whether potential alternative tick vectors are infected with *T. parv*a.

*Theileria parva* is not currently endemic in Cameroon; therefore, confirmed data on ECF outbreaks is scarce although there are recent reports of animals with symptoms similar to those observed in theileriosis (R. Pelle and B. A. Silatsa personal communication). This lack of information does not necessarily mean that animals with clinical disease are completely absent from the country. Since the diseases are not present on the list of known infections, veterinary service personnel are not trained to diagnose ECF in the field. Furthermore, currently the majority of infected cattle are subclinical carriers, which may never progress to full disease status. Because the currently known ECF vectors (*R. appendiculatus*, *R. zambiensis* and *R. duttoni*) have not been detected in Cameroon during recent surveys (Silatsa, Simo, et al., 2019b), the infected animals do not currently pose any risk of infection to naive cattle, although this situation could change in future, since certain AEZs, such as the Adamawa plateau, the western highlands and the coastal belt are climatically suitable for establishment of the major vector *R. appendiculatus* (Walker et al., 2003).

The clinical outcome of an infection is affected by many factors, among which are the history of infection by another parasite, and current co‐infection with a related parasite. It has been demonstrated that co‐infection of *T. parva* with other parasites, particularly prior infection with *T. mutans,* frequently results in reduced severity of subsequent *T. parva* infections (Van Wyk et al., 2014; Woolhouse et al., 2015). In our study, co‐infection of *T. parva* and *T. mutans* appeared to be low, although apparent prevalence of *T. mutans* according to serology and RLB was high (40%–80%), and *T. mutans* was detected in 98.3% of *T. parva* RLB positive samples. Although serology does not indicate whether *T. mutans* is still present in an animal, RLB indicates active infection with *T. mutans* which are transmitted by ticks of the genus *Amblyomma*. A recent study on ticks infesting cattle in Cameroon ranks *Amblyomma variegatum* as the most prevalent and ubiquitous tick species in Cameroon (Silatsa, Simo, et al., 2019b). Therefore, we hypothesise that *T. mutans* is actively transmitted in Cameroon, due to the combined presence of host, vector and pathogen and that severe ECF is not observed in infected cattle, at least in part, because *T. mutans* infection decreases the pathology associated with *T. parva* infection. As previously suggested by Woolhouse et al. (2015), it is possible that infections with *T. mutans* could provide heterologous protection against *T. parva*, contributing to the subclinical nature of *Theileria parva* infection in cattle in Cameroon. It is also possible that some *T. parva* infected cattle that migrated into Cameroon from adjacent countries, including South Sudan and the Central African Republic, had recovered naturally from exposure to *T. parva* and remained persistently infected, since there have been reports of a *T. parva* carrier state in areas where tick challenge in the field is relatively low and the animals are of *Bos indicus* genotype (Young, Leitch, & Newson, 1981).

*Theileria mutans* infections were more frequently detected than *T. parva* infections using both serology and RLB. These findings are in agreement with previous studies using the same techniques (Byaruhanga et al., 2016; Kiara et al., 2014; Njiiri et al., 2015). As previously mentioned, this difference could be explained by more frequent transmission of *T. mutans* in the field, by contrast with *T. parva* for which the main vector is apparently absent in Cameroon (Silatsa, Simo, et al., 2019b), and for which transmission still needs to be demonstrated, although it seems likely based on the 23% *T. parva* seroprevalence such transmission may occur. Furthermore, unlike *T. parva*, *T. mutans* undergoes limited lymphocytic merogony. Therefore, the main site of replication for *T. mutans* occurs in erythrocytes, which results in a higher piroplasm parasitemia for *T. mutans* infections compared to *T. parva* and therefore greater sensitivity of RLB for *T. mutans* relative to *T. parva* (Oura, Bishop, Wampande, Lubega, & Tait, 2004) Furthermore, it has been demonstrated that during *T. parva* infections, parasitemia fluctuates above and below the limit of detection using PCR (Bishop et al., 1992; Odongo et al., 2010; Skilton et al., 2002). By contrast, *T. mutans* seroprevalence was lower than prevalence assessed using RLB. This could be explained by the fact that antibody responses to *T. mutans* last only for a relatively short period of time and are not sustained as compared to other haemoparasites (Kiara et al., 2014).

p104 amino acid sequences analysis of 18 *T. parva* isolates from Cameroon revealed seven variants. 39% of the variants were different from previously described *T. parva* sequences generated from this locus (Iams et al., 1990; Skilton et al., 2002). These findings suggest that *T. parva* isolates from Cameroon are more diverse than previously revealed from a limited sample of isolates. Given the absence of the main tick vector of *T. parva* in Cameroon, this high level of diversity cannot be explained by a high level of transmission and recombination of parasites strains in the tick vector. Instead, the diversity could be explained by the occurrence of multiple events of importation of different genotypes of *T. parva* from East Africa. This scenario has been previously described in South Sudan (Salih, El‐Hussein, Marcellino, Berkvens, & Geysen, 2015).

It has been demonstrated that cattle in contact with buffalo harbour more diverse *T. parva* parasite populations than cattle from populations that have no recent contact with buffalo (Oura et al., 2003). A recent study of cattle husbandry and dairy practices among pastoralists and small‐scale farmers in Cameroon indicates that 25% of the herds in the population studied had potentially contacted buffalo during transhumance (Kelly et al., 2016). This could provide an alternative explanation for the diversity observed in *T. parva* strains from Cameroon.

Based on p104 gene analysis, 61% of *T. parva* isolates from Cameroon were identical to alleles 1 and 2 which were first described in the *T. parva* Muguga and Marikebuni stocks used as vaccines and commercialized in Kenya (Bishop et al., 2001; Payne, 1999).With regard to presence of *T. parva* infections in cattle from Cameroon, it is possible that at least some of these infections could be result of anthopogenically mediated movement of animals, immunized using the live ITM vaccine. This hypothesis is strengthened by the fact that the Tp1 CD8 epitope present in the Cameroonian animals is identical to that observed in all three components of the Muguga cocktail. Transnational transhumance and conflicts in the subregion result in Cameroon being epidemiologically connected to eastern and central Africa countries including South Sudan, Democratic Republic of Congo and Uganda where ECF is endemic (Luizza, 2017; Seignobos, 2008). It seems most likely that the *T. parva* infections observed in cattle in Cameroon are currently primarily a result of transboundary movement of animals that have been naturally infected or were vaccinated by ITM. Recently, we demonstrated that CD8 T cell alleles found in the Muguga Cocktail ITM vaccine are present in at least 70% of *T. parva* populations in the field (Pelle et al., 2011). In addition, p104 primers are pan‐specific and can therefore detect many *T. parva* isolates, including those from buffalo. Therefore, it is currently unclear whether infections described in this study originated from cattle vaccinated by ITM or naturally infected cattle. Given that most large scale ITM vaccination has been in Tanzania and Southern Kenya (Di Giulio et al., 2009), the latter scenario seems more likely. The molecular diversity of the parasite at the p104 locus, suggests that there could have been multiple sources of *T. parva* introduction. This creates a very dangerous scenario in which introduction, or evolution of efficient *T. parva* vectors, could lead in a major extension of the ECF endemic region into central Africa.

## 5 CONCLUSION

This study revealed for the first time *T. parva* infections within cattle from Cameroon and provided initial insights into the diversity of the genotype present in country. The study highlighted similarities of *T. parva* isolates from Cameroon to most isolates found in the field in ECF endemic regions as well as to the stocks used as components of the Muguga Cocktail live vaccine increasingly deployed in eastern Africa. We speculate that cross‐border cattle movement has imported the infection into the country. There is as yet no evidence that tick transmission of the parasite is occurring in Cameroon, but this possibility cannot be entirely excluded. Further studies are will be required to determine the infection rates of *T. parva* in ticks. The present study has revealed the requirement for in‐depth surveillance and monitoring of cattle with theileriosis‐like symptoms in the country in order to facilitate rapid control of possible future ECF outbreaks in Cameroon.

## CONFLICT OF INTEREST

The authors declare that they have no competing interests.

## ETHICAL APPROVAL

Permission to undertake the study was obtained from the Ministry of Livestock, Animal Husbandry and Fisheries reference number 026/L/MINEPIA/MSEG. The farmers provided signed informed consent for the use of their cattle and information for the study. The collection of blood on cattle did not involve national parks or other protected areas or endangered or protected species.
